# Supplementary material for: Cu-BTC Derived Mesoporous CuS Nanomaterial as Nanozyme for Colorimetric Detection of Glutathione
Source: Molecules. 2024 May 3;29(9):2117. doi: 10.3390/molecules29092117 (PMC11085296; doi:10.3390/molecules29092117)
Supplement: Supplementary file 1 [file molecules-29-02117-s001.zip › molecules-2950622-supplementary.pdf]

Supporting Information for Cu-BTC Derived Mesoporous CuS Nanomaterial as Nanozyme for Colorimetric Detection of Glutathione

Xiwen Zhang [1†], Jie Wang[2, 3†], Nan Chang[4], Yu Yang[5], Yuqi Li[3], Qi Wei[3], Chang Ni[3], Wanying Song[3], Mingyue Ma[6], Xun Feng[7*] and Ronghua Fan[3*]

1. School of Basic Medicine, Shenyang Medical College, Shenyang 110034, China
2. Department of Science and Technology, Shenyang Medical College, Shenyang 110034, China
3. Department of Sanitary Inspection, School of Public Health, Shenyang Medical College, Shenyang 110034, China
4 . Department of Food Science, School of Public Health, Shenyang Medical College,Shenyang 110034, China
5. Department of Physiology, School of Basic Medicine, Shenyang Medical College, Shenyang 110034, China
6. Department of Toxicology, School of Public Heath, Shenyang Medical College, Shenyang 110034, China
7. Department of Sanitary Chemisrty, School of Public Health, Shenyang Medical College, Shenyang 110034, China

*Correspondence: ronghuafan1@126.com (R.F.); fengxundfx@163.com (X.F.)
Tel.: +86-135-1603-5668 (R.F.) ; +86-159-4208-6933 (X.F.)

† These authors contributed equally to this work

Dr. Ronghua Fan
Department of Sanitary Inspection, School of Public Health
Shenyang Medical College, Shenyang 110034, China
E-mail: ronghuafan1@126.com
Dr. Xun Feng
Department of Sanitary Chemisrty, School of Public Health
Shenyang Medical College, Shenyang 110034, China
E-mail:fengxundfx@163.com

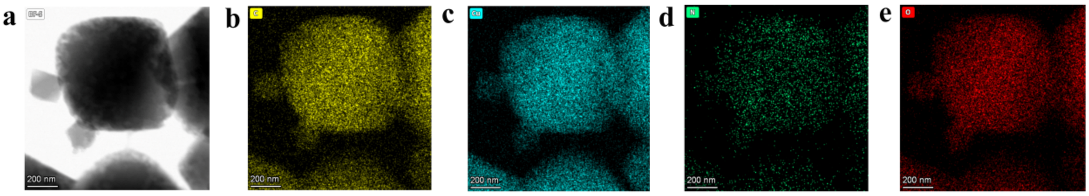

Figure S1 EDX mapping of Cu-BTC. (a) TEM of Cu-BTC, (b) C element, (c) Cu element, (d)N element, (e) O element.

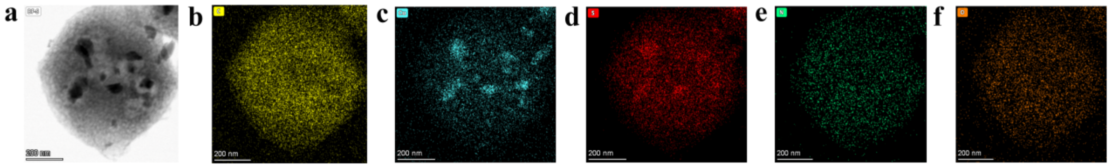

Figure S2 EDX mapping of m-CuS. (a) TEM of m-CuS, (b) C element, (c) Cu element, (d) S element (e)N element, (f) O element

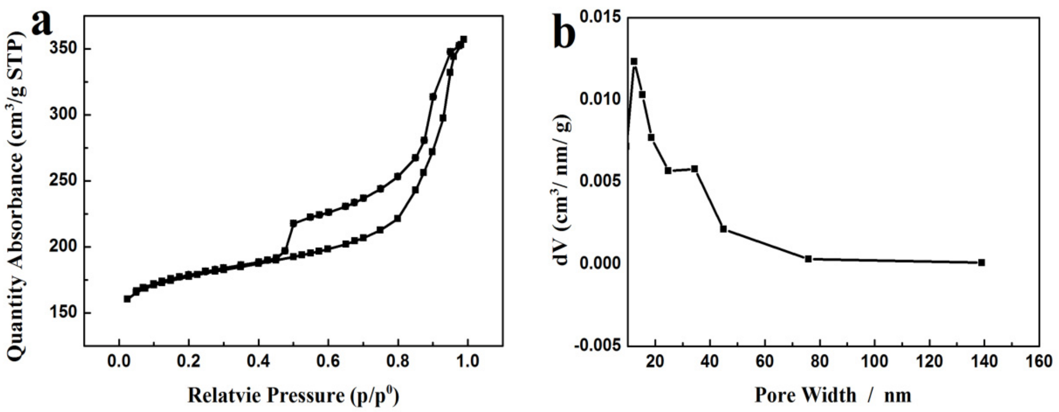

Figure S3 (**a**) Nitrogen adsorption-desorption isotherm; (**b**) Pore size distribution of the Cu-BTC

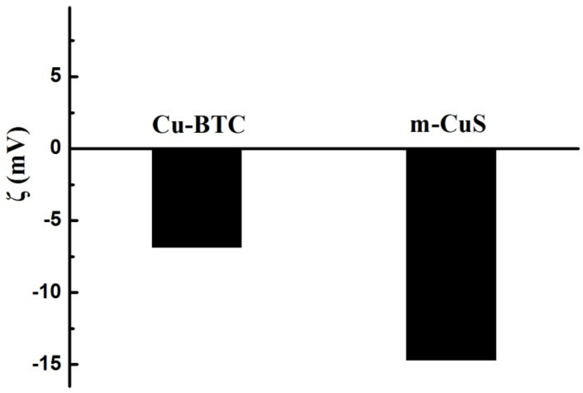

Figure S4 Zeta potential values of Cu-BTC and m-CuS
